# Potential Risks for Seahorse Stock Enhancement: Insight From the Declivity of Genetic Levels With Hatchery Management

**DOI:** 10.3389/fgene.2021.830626

**Published:** 2022-01-20

**Authors:** Wei Luo, Qing Wu, Xiaoyang Zhang, Yuling Wei, Min Liao, Tong Gao, Yibo Zhang, Shoudong Zhang, Pengyu Chen, Zhonggang Guo, Yinlin Xiong, Zhou Xu, Zongjun Du

**Affiliations:** ^1^ College of Animal Science and Technology, Sichuan Agricultural University, Chengdu, China; ^2^ Chongzhou Agricultural and Rural Bureau, Chengdu, China; ^3^ The Original Stock Farm of Leiocassis Longirostris of Sichuan Province, Chengdu, China; ^4^ Mianyang Academy of Agricultural Sciences, Mianyang, China

**Keywords:** stock enhancement, genetic diversity, population genetics, genetic risk, seahorse

## Abstract

Stock enhancement is one of the potential management strategies for the fishery. To better understand the impaction of stock enhancement, we simulated an experiment for lined seahorse (*Hippocampus erectus*) and evaluated the genetic structure after stock enhancement. In this study, we found the numbers of alleles (*N*_*A*_) and heterozygosity (*H*_*O*_) of stock enhancement strains were lower than those of the wild collections, while the inbreeding coefficient (*F*_*IS*_) and relatedness index were higher. Within the 3 generations of stock enhancement strain, the *N*_*A*_, *H*_*O*_ and polymorphism information content (*PIC*) didn’t change significantly. In addition, the *F*_*ST*_ value indicated that the genetic differentiation between the stock enhancement strains and the first wild collection reached an intermediate level, which could lead to substructuring in wild populations. Overall, these findings revealed a potential genetic risk associated with the release of hatchery strains into wild populations.

## Introduction

Recent studies show that the living conditions of most wild commercial and protected fisheries have been altered by overfishing, pollution and degradation of habitats, and leading to the decline of the stocks (Hansen, 2002). Supporting weak wild populations through the release of conspecifics (stock enhancement) is being used increasingly in conservation practice. More than 65 marine and brackish-water species have been extensively subjected to stocking practices across 27 countries with the goal of increasing exploitable resources (Bartley and Bell, 2008).

Stock enhancement has been used for more than 100 years; however, its efficacy and risk to natural populations has been controversial (Stottrup and Sparrevohn, 2007; Araki and Schmid, 2010). In particular, hatchery often leads to genetic, morphological and behavioral differences between captive-bred and wild populations due to environmental differences, and human intervention (Svasand et al., 2000; Araki et al., 2007; Blanchet et al., 2008). Genetic effects of hatchery programs on wild populations have been investigated in many species, but the effects affect the ecology, genetics, and fitness of the wild stocks is still not clear (Araki and Schmid, 2010; Hold et al., 2013). Some researchers did not find any direct evidence for the genetic effects of hatchery fish on wild populations, such as in sea trout (*Salmo trutta*) (Palm et al., 2003), *G. morhua* (Svasand et al., 2000), channel catfish (*Ictalurus punctatus*) (Simmons et al., 2006). Conversely, other researchers reported that hatchery fish have obvious genetic effects on wild populations, such as in Atlantic salmon (Skaala et al., 2006), coho salmon (Eldridge and Naish, 2007). In such cases, evaluating the utility of captive breeding for stock enhancement considering the associated genetic risks is a prerequisite for future management decisions.

Seahorses, genus *Hippocampus*, are highly unusual marine fishes with unique body morphology, and specialized life history traits. Their biological and ecological characteristics, including low mobility, small home ranges, sparse distribution, male pregnancy, lengthy parental care and strict monogamy in most species, might render them vulnerable to overfishing or habitat damage (Foster and Vincent, 2004; Curtis and Vincent, 2006). Worldwide, many seahorse species are considered to be under threat of overexploitation for traditional medicines, aquarium trade and ornaments (Foster and Vincent, 2004; Koldewey and Martin-Smith, 2010). The entire genus *Hippocampus* is listed on Appendix II of the Convention on International Trade in Endangered Species of Wild Fauna and Flora (CITES), and the majority of seahorse species are listed as data deficient with several others listed as vulnerable or endangered (Pollom, 2017).

In this study, we simulated a stock enhancement and evaluated the impact of hatchery rearing on the genetic structure of wild seahorse populations using eleven microsatellite markers, with the purpose of providing a scientific basis for wild resource recovery for seahorse in the future.

## Materials and Methods

### Study Populations

**FIGURE 1:**
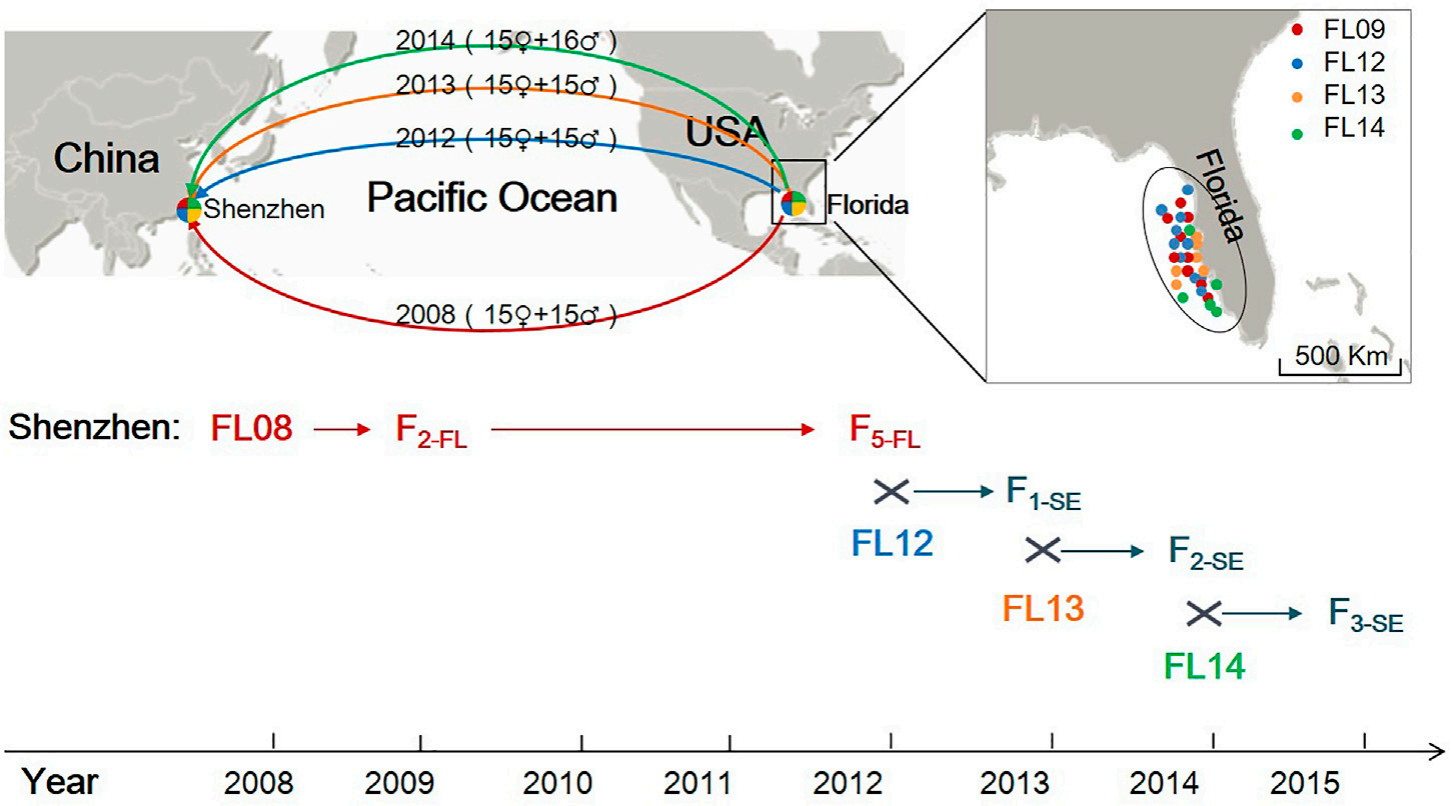
The sampling sites of wild lined seahorses, a sketch of the introduction from the United States to China and a flowchart of the hatchery and a simulated stock enhancement in China. Red, blue, orange, and green lines indicate the introduction from Florida to Shenzhen in 2008, 2012, 2013, and 2014, respectively.

Four collections of the lined seahorses (FL08, FL12, FL13, and FL14) were sampled from the region of the west coast of Florida (United States) in 2008, 2012, 2013, and 2014, respectively (Figure 1). The seahorses were cultured in separate concrete outdoor ponds (3 × 3 × 1 m), with recirculating sea water treated with double sand filtration. Seahorses were fed daily with rotifers, copepods, *artemia*, *Mysis* spp., and *Acetes* spp. The FL08 collection, as initial breeders, was used for artificial breeding. Their fry (F_1-FL_) were immediately transferred to another tank after they were hatched out. After about 6 months of feeding, they reached maturity and 30 individuals (sex ration 1:1) were randomly selected for reproducing the next generation. In a similar fashion, offspring of the second to the fifth generation of FL08 were obtained. In 2012, to simulate the influence of stock enhancement in releasing the hatchery strains into the wild, we designed a mating between the FL12 collection and the fifth generation of FL08 (F_5-FL_). We randomly selected 30 individuals of F_5-FL_ (sex ration 1:1) and put them together with FL12 collection (sex ration 1:1) in a culture pond. In 2013 and 2014, we did a mating between F_1-SE_ (randomly selected) and FL13 and F_2-SE_ (randomly selected) and FL14, respectively. In this study, we assumed that the “stock enhancement” strains (named F_i-SE_, where *i* is the generation) were mated with wild individuals with number ratio of 1:1 each generation in the simulation. In this study, thirty individuals of each collection, including 4 wild collections, 2 hatchery strains, and 3 ″stock enhancement” strains were sampled for genetic analysis.

### DNA Extraction, Amplification and Microsatellite Analysis

Genomic DNA was extracted from approximately 100 mg of dorsal fin tissue from every sample by standard phenol-chloroform protocols. All the samples were genotyped with eleven microsatellite markers, of which *Hier-ssr3*, *Hier-ssr7*, *Hier-ssr8*, *Hier-ssr9*, *Hier-ssr10*, *Hier-ssr13*, *Hier-ssr15*, *Hier-ssr17*, *Hier-ssr28,* and *Hier-ssr29* were previously developed by our group (Luo et al., 2015; Arias et al., 2016), and *Hier-ssr51* was designed in this study (Supplementary Table S1). The method of PCR amplification and microsatellite analysis referred to Luo et al. (2017).

### Genetic Variation Analysis

The number of alleles (*N*_*A*_), the effective number of alleles (*N*_*AE*_), allele frequency (*AF*), observed heterozygosity (*H*_*O*_), expected heterozygosity (*H*_*E*_), the genetic distance (GD), and the departure from Hardy-Weinberg equilibrium (*HWE*) of each locus were calculated using POPGENE32 (ver. 1.32). A UPGMA phylogenetic tree was built using MEGA 5.1 software according to the information on *GD* between different populations. Analysis of molecular variance (AMOVA) of the genetic structure, the pairwise coefficient of genetic differentiation (*F*_*ST*_), the coefficient of inbreeding (*F*_*IS*_) among the ten populations, and the presence of null alleles were estimated using Arlequin software (ver. 3.11). The relatedness index among the individuals sampled was calculated using the method of TrioML by Coancestry software.

## Results

### Genetic Diversity of Wild, Hatchery and “Stock Enhancement” Strains

A total of 103 alleles at all loci were detected in the 4 wild collections with a mean value of 9.36 per locus, and there was no obvious change of *N*_*A*_ within the 4 collections. The mean *N*_*A*_ value of first hatchery strain generation of FL08 (F_1-FL_) was 8.18 per locus, and that of the fifth generation (F_5-FL_) declined to 8.00. The *N*_*A*_ of the first generation of stock enhancement strains (F_1-SE_) recovered to 8.55, and that of the second (F_2-SE_) and the third generation (F_3-SE_) of stock enhancement strains was a little lower. Similar to *N*_*A*_, the *N*_*AE*_ values of the hatchery strains gradually declined over generations, and that of the 3 “stock enhancement” strains slightly recovered, but it was still lower than that of wild collections (Supplementary Table S2).

No null alleles were detected in the hatchery strains and stock enhancement strains. Linkage disequilibrium was assessed and no significant departure from equilibrium levels was detected in any sample. No locus was significantly departed from *HWE* (*p* < 0.05) in the 4 wild collections, while 1, 2, and 2 were significantly departed from *HWE* (*p* < 0.05) in F_1-FL_, F_2-FL_, and F_5-FL_ and 1, 1 and 2 were in F_1-SE_, F_2-SE_, and F_3-SE_, respectively. All significant deviations from Hardy–Weinberg expected heterozygosities were due to an excess of homozygotes. Compared with the genotype data of the FL08 and F_5-FL_ collections, some alleles, especially rare alleles of *Hier-ssr7*, *Hier-ssr8*, *Hier-ssr9,* and *Hier-ssr10*, were found to be lost in the process of captive breeding. Similar to the hatchery strains, some alleles of *Hier-ssr9* and *Hier-ssr10* were also lost in the “stock enhancement” strains, but some alleles, such as those of *Hier-ssr15*, and reappeared in these strains (Supplementary Table S2).

The minimal allele frequency (*MAF*) exhibited a growing disparity with generations due to allele loss; some alleles were gradually reduced until they disappeared, and some gradually increased and dominated the population. The mean *MAF* values of the 4 wild collections over all loci were 0.023, 0.026, 0.029, and 0.021; while those of the hatchery strains and “stock enhancement” strains increased, especially for F_5-FL_, with mean values of 0.036.

**FIGURE 2:**
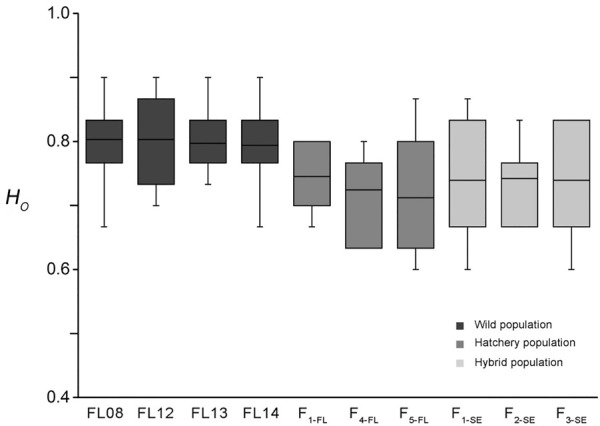
The box plot of *H*_*O*_ values of wild (FL08, FL12, FL13, and FL14), hatchery (F_1-FL_, F_4-FL_, and F_5-FL_) and “enhancement stock” strains (F_1-SE_, F_2-SE_, and F_3-SE_) of lined seahorse.

The mean value of *H*_*O*_ of the FL08 collection over all loci was 0.80, with a minimum of 0.67 at locus *Hier-ssr38* and a maximum of 0.90 at locus *Hier-ssr26* and *Hier-ssr15*. The mean values of *H*_*O*_ of other 3 wild collections were similar with that of FL08. While that of F_1-FL_, F_4-FL_, and F_5-FL_ was 0.75, 0.72, and 0.71, respectively. The mean values of *H*_*O*_ of the 3 ″stock enhancement” strains were all about 0.74, which were higher than that of F_5-FL_ but lower than those of wild collections (Figure 2). The value of *H*_*E*_ of wild collections ranged from 0.80 to 0.81, while that in hatchery strains from 0.75 to 0.76 and “stock enhancement” strains from 0.74 to 0.76 (Supplementary Table S2).

The mean *PIC* values of all loci in the 4 wild collections were closed to 0.76. While, the mean *PIC* values of F_1-FL_, F_4-FL_, and F_5-FL_ strains was 0.71, 0.69, and 0.69, respectively. The *PIC* values of “stock enhancement” strains ranged from 0.71 to 0.74, which were higher than those of hatchery strains but lower than those of wild collections (Supplementary Table S2).

**FIGURE 3:**
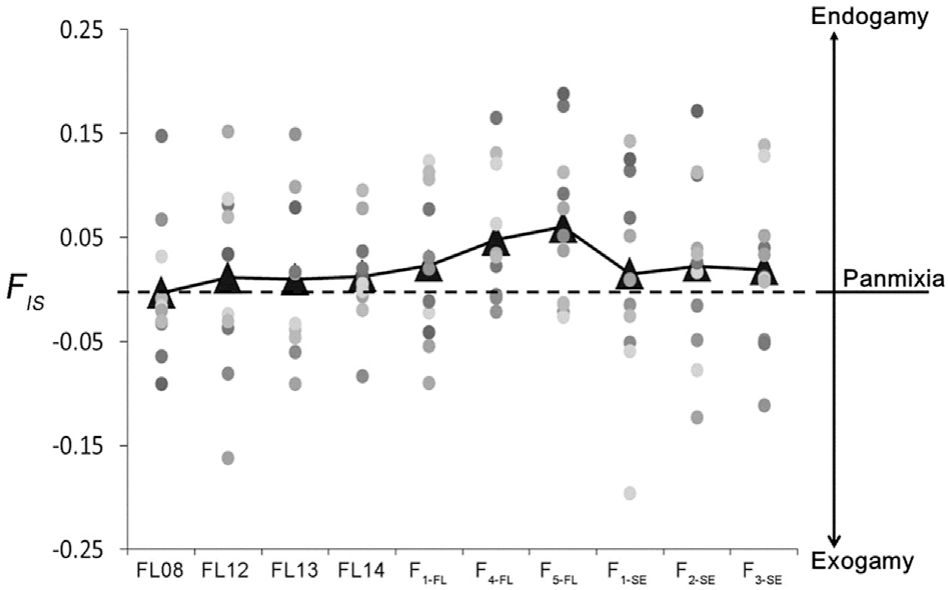
The inbreeding coefficient (*F*_*IS*_) of lined seahorse collections (4 wild collections, 3 hatchery strains and 3 “stock enhancement” strains). Dots with different colors represent different microsatellite loci. Triangles represent the mean values of *F*_*IS*_ of different collections.

The inbreeding coefficient (*F*_*IS*_) was close to 0 for the 4 wild collections. The *F*_*IS*_ values of the 3 hatchery strains were higher than those of wild collections, and the inbreeding rate gradually accumulated over generations with highest *F*_*IS*_ value of 0.06 in the F_5-FL_ strain. In addition, the *F*_*IS*_ values of all the 3 ″stock enhancement” strains maintained about 0.02 (Figure 3), indicating there existed a small risk of inbreeding in the wild after stock enhancement. The mean relatedness index of the wild collections was from 0.067 to 0.070; while that of the hatchery stains was higher and increased over generations (the relatedness index of F_5-FL_ reached to 0.106). The mean relatedness index of “stock enhancement” strains was lower than that of hatchery stains but higher than that of wild collections (Appendix A).

### Genetic Divergence Among Wild, Hatchery and “Stock Enhancement” Collections

**FIGURE 4:**
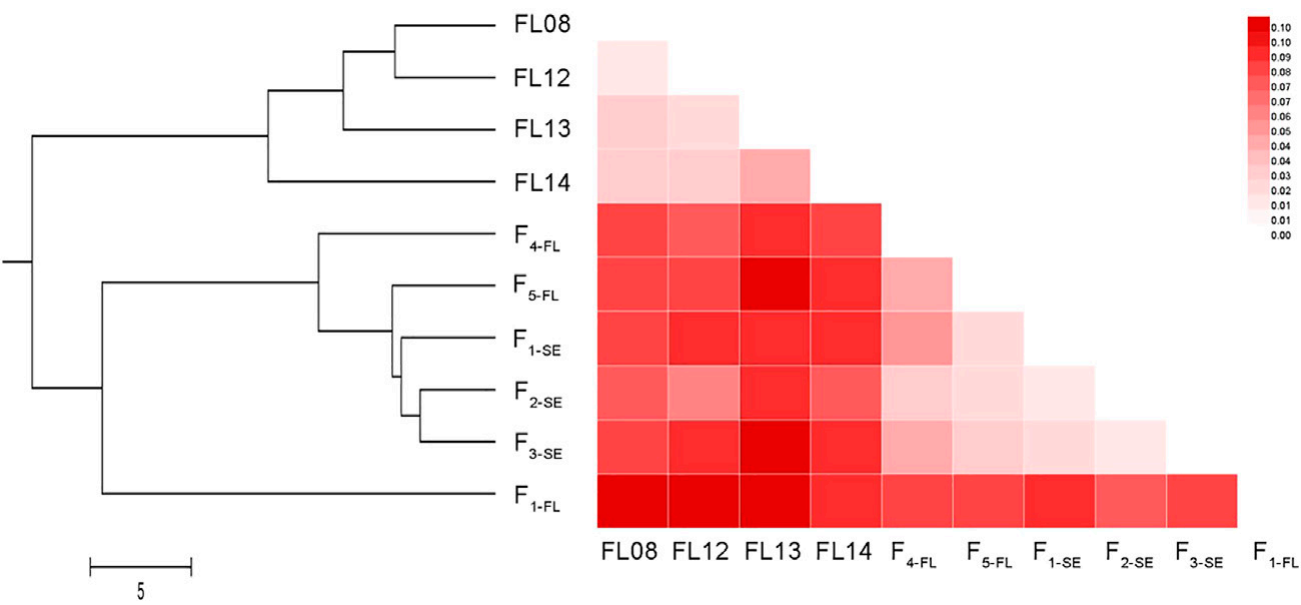
The UPGMA phylogenetic tree and heatmap of *F*_*ST*_ values among 10 lined seahorse collections. UPGMA clustering was based on *GD* among pairs of populations and the tree was built using MEGA 5.1 software.

The range of *GD* of the ten collections was from 0.08 (the pair of F_1-SE_ and F_2-SE_) to 0.57 (the pair of F_2-SE_ and FL12). The UPGMA phylogenetic tree based on *GD* among pairs of populations (Figure 4) was divided into two branches: one represents the 4 wild collections, and the other represents the hatchery strains and “stock enhancement” strains. In the lower branch, the 3 “stock enhancement” strains were clustered together firstly, and then F_1-FL_, F_4-FL_, and F_5-FL_ was separated from the branch of “stock enhancement” strains successively.

The value of *F*_*ST*_ ranged from 0.02 (the pair of FL08 and FL13) to 0.11 (the pair of FL12 and F_1-SE_) for all collections (Figure 4). The *F*_*ST*_ values between F_3-SE_ and FL08, FL12, FL13, and FL14 were 0.11, 0.11, 0.10, and 0.09, respectively, indicating a medium level of genetic differentiation between “stock enhancement” strains and wild populations. The result of AMOVA of all ten collections revealed that 92.65% of genetic differentiation was within populations, and only 7.35% was among collections (Supplementary Table S3).

## Discussion

In aquaculture and wildlife protection, understanding whether genetic variability is lost in cultured stocks after long periods of hatchery production is important, because reduction in variability may possibly result in the loss of genetic variation for disease resistance and reduce a population’s capability to adapt to new environments (Li et al., 2004). Unfortunately, reduction in allelic richness, which was found in many reported cultured species, including fish, shrimp and shellfish (Li et al., 2004; Kohlmann et al., 2005; Marchant et al., 2009), and seems to be a characteristic of hatchery populations. There is no exception in the captive breeding of seahorse. The results showed that the values of *N*_*A*_, *H*_*O*_, and *PIC* of the 3 hatchery strains were lower than those of wild collections, and the impact of hatchery rearing on the loss of allele richness seemed to be more and more serious over generations. Specifically, the F_5-FL_ strain lost approximately 13% of the number of alleles and 11% of the heterozygosity compared to FL08 collection, respectively. In addition, we found the mean relatedness index of hatchery strains was higher than that of wild collections, especially some pairs of hatchery strains had high relatedness value. Serial founder effects across generation and associated genetic drift could be the major factor responsible for the reduction of diversity of hatchery strains. Moreover, unequal contribution to offspring of breeders due to artificial selection in the hatchery process, may lead to a reduction of some alleles and even the disappearance and increase of the effect of genetic drift (Nakajima et al., 2014).

The reduction in genetic diversity and changes in allelic frequencies of hatchery strains may pose potential genetic risks to wild populations in supportive breeding programs (Machado-Schiaffino et al., 2007). The introduction of progeny with low genetic diversity into wild seahorse populations has led to lowered overall genetic variability of the resulting populations (Utter, 1998). In our study, the values of *N*_*A*_, *H*_*O*_, and *PIC* of the 3 “stock enhancement” strains were lower, but *MAF* were higher than those of wild collections. In addition, the allelic richness and heterozygosity of “stock enhancement” strains did not rise within 3 generations after seahorse being released into wild, indicating consecutive interbreeding with wild-collected seahorses failed to reintroduce lost alleles or genetic diversity. The reduction of genetic diversity in wild population seems to persist for a long time. Specially, seahorse has high mate fidelity and lengthy parental care (Foster and Vincent, 2004; Koldewey and Martin-Smith, 2010), restricting multiple mating between individuals during their lifespan; they also have low mobility and small home ranges (Curtis and Vincent, 2006; Koldewey and Martin-Smith, 2010), limiting gene flow amongst wild populations. Thus, their specialized life history traits may make seahorse populations particularly susceptible to anthropogenic disturbances, and their genetic diversity is easy to reduce and difficult to recover (Foster and Vincent, 2004; Curtis and Vincent, 2006). Our findings are in accordance with numerous previous reports on stock enhancement of fish species, such as brown trout (*Salmo trutta*) (Hansen, 2002), red sea bream (*Pagrus major*) (Kitada et al., 2009), and Pacific herring (*Clupea pallasii*) (Sugaya et al., 2008).

In this study, some alleles were lost and the allele frequency changed a lot in the hatchery process, which lead to an increase in the mean value of *MAF*, and a significant departure from *HWE* for some loci in the hatchery and “stock enhancement” strains. A previous study reported on pacific abalone (*Haliotis discus hannai*) that random changes in allele frequencies could be caused by genetic drift in a depleted or small hatchery population (Li et al., 2004). The non-random mating system has changed the overall allelic composition of the farmed strain relative to the wild population, and the overall allelic composition of the “stock enhancement” strains might not be possible to restore to the original form (Li et al., 2004; Kitada et al., 2009; Hold et al., 2013). The concern is that declines in variation at neutral markers such as these may be indicative of a loss of variation at coding regions, which is the source of variation in important commercial traits such as growth rate and disease resistance.

Selection that occurs in hatchery rearing could be deleterious because traits that are advantageous in the reared environment may be disadvantageous in the wild. In other words, if the trait distribution in a wild population is at an optimum that has been shaped by selection in the wild environment, releasing individuals into the population that have a different distribution as a result of selection in a hatchery will result in a reduction in the mean fitness of the population (Araki et al., 2007; Kitada et al., 2009; Jonsson et al., 2016). Factors such as predation, escape, competition and stress tolerance are less important under culturing environments with adequate food, less competition, a steady water environment, and a homogeneous habitat in the long-term process of hatching (Stottrup and Sparrevohn, 2007; Kitada et al., 2009). Experience with salmonid culture shows that, with or without intentional selection of parents, each successive generation of captive brood stock will favor those parents and progeny that are most amenable to conditions of culture (Utter, 1998); while, in the wild environment, populations have evolved different life-history strategies, which can be attributed to interior intra-species interactions, and environment heterogenetity (Svasand et al., 2000). A potential risk associated with inbreeding depression could be brought into the natural ecosystem because of high inbreeding rate and relatedness value accumulated over generations in hatchery rearing. Even after serial generations of mating with wild individuals, our result demonstrated the “stock enhancement” strains still kept medium inbreeding rate and relatedness. The phenomenon was also found in most cultured species, such as abalone (*Haliotis discus hannai*) (Marchant et al., 2009) and common carp (*Cyprinus carpio* L.) (Kohlmann et al., 2005). The tendency towards homozygosity could be explained by the founder effect (Marchant et al., 2009).

Recent studies demonstrated that repeated interbreeding between a natural population and mass-released hatchery strains could result in a non-random mating system or, possibly, assortative mating that could generate within-population substructuring (Wahlund effect), and inbreeding (Sekino et al., 2005). In this study, the pairwise *F*_*ST*_ and *GD* values indicated a medium genetic differentiation between “stock enhancement” strains and wild collections (According to Wright’s criteria (0 < *F*_*ST*_ < 0.05, no differentiation; 0.05 < *F*_*ST*_ < 0.15, medium level; and 0.15 < *F*_*ST*_ < 0.25, high level). The results of AMOVA supported relatively high genetic variation within collections. The genetic differentiation between “stock enhancement” strains and wild collections was medium, which poses a genetic risk of within-population substructuring to the natural populations (Sekino et al., 2005).

Although a potential risk exists, many measures can be used to mitigate or remove the risk for seahorse stock enhancement. Introducing large numbers of wild breeders into hatchery could be one of the most effective ways to maintain the genetic diversity of hatchery populations (Kitada et al., 2009). Moreover, it is necessary for us to maintain clear genetic lineage by building marker-based pedigree assignment and to design a mating strategy based on genetic distance to avoid inbreeding depression in seahorse hatcheries.

In conclusion, the results of this study suggested that the genetic diversity in terms of allelic diversity and heterozygosity decreased but the inbreeding coefficient increased over generations in the hatchery compared to wild collections. Moreover, the genetic diversity of “stock enhancement” collections was lower than the wild population and it seemed that consecutive interbreeding with wild-collected seahorses did not improve the genetic diversity very well. In addition, the genetic differentiation between wild and “stock enhancement” collections reached a medium level, and which could lead to substructuring in wild populations. Considering the highly unique life history traits of the seahorse, these findings reveal a potential risk associated with the release of hatchery fishes into wild populations. This study provides valuable information on seahorse resource conservation and recovery from a genetic perspective.

## Data Availability

The datasets presented in this study can be found in online repositories. The names of the repository/repositories and accession number(s) can be found in the article/Supplementary Material.
