# Supplementary material for: Potential Risks for Seahorse Stock Enhancement: Insight From the Declivity of Genetic Levels With Hatchery Management
Source: Front Genet. 2022 Jan 20;12:830626. doi: 10.3389/fgene.2021.830626 (PMC8811164; doi:10.3389/fgene.2021.830626)
Supplement: Supplementary file 1 [file Table2.DOCX]

**Table S1** The primers information of the 11 microsatellite markers of the lined seahorses (*Hippocampus erectus*) used in this study

| **Primer ID** | **Primer sequences (5’-3’)** | **Size (bp)** | **Tm (°C)** |
| --- | --- | --- | --- |
| Hier-ssr3 | F:ACCTGCTACCAACCACAA  R:AACAAGGCAACCACTCATT | 271–285 | 54 |
| Hier-ssr7 | F:TGAGTCGGTGATGGTTGTG  R:AAAGACGGCGAGAGATAGG | 100-112 | 58 |
| Hier-ssr8 | F:TGATGGTTGTGTGAGAAGGA  R:GAGAGAAAAAGACGGCGA | 121-149 | 58 |
| Hier-ssr9 | F:GTTGATGCGACCCACGAT  R:GCCTTGCTCCTTCTCTACG | 220-242 | 56 |
| Hier-ssr10 | F:CCAGTTAGCATTGCGTCT  R:TCTTAGCCAGCGAGTGTT | 104-116 | 54 |
| Hier-ssr13 | F:TGCCATCATCGCTAACTAA  R:TGCCAAAGACACAAAAAGG | 206-222 | 54 |
| Hier-ssr15 | F:AGTGGGTGTCTCTGTAAAC  R:CCTTCGCAGTATTCATTG | 186-198 | 56 |
| Hier-ssr17 | F:TGCCGAATGATGATACAC  R:AATGACGCAATGAGAACA | 230-246 | 52 |
| Hier-ssr28 | F:GTGAGAAACGCACCAAAC  R:ACACGACAAAACAGCATC | 137-155 | 54 |
| Hier-ssr29 | F:TGCTCACAGGCTTCACAT  R:TCCTTTTCACAGTCCCAC | 203-223 | 54 |
| Hier-ssr51 | F:CTCAGCACCACGCACTCC  R:CTCAGCACCACGCACTCC | 166-178 | 60 |
