# Supplementary material for: Potential Risks for Seahorse Stock Enhancement: Insight From the Declivity of Genetic Levels With Hatchery Management
Source: Front Genet. 2022 Jan 20;12:830626. doi: 10.3389/fgene.2021.830626 (PMC8811164; doi:10.3389/fgene.2021.830626)
Supplement: Supplementary file 2 [file Table3.DOCX]

**Table S2** Genetic variability indices of lined seahorse (*Hippocampus erectus*) populations

| **Locus** | **Genetic variability indices** | **Wild collections** | | | | **Hatchery strains** | | | **"stock enhancement" strains** | | |
| --- | --- | --- | --- | --- | --- | --- | --- | --- | --- | --- | --- |
|  |  | FL08 | FL 12 | FL 13 | FL14 | F_1-FL_ | F_4-FL_ | F_5-FL_ | F_1-SE_ | F_2-SE_ | F_3-SE_ |
|  |  | (n = 30) | (n = 30) | (n = 30) | (n = 31) | (n = 30) | (n = 30) | (n = 30) | (n = 30) | (n = 30) | (n = 30) |
| *Hier-ssr3* | *N_A_* | 9 | 10 | 10 | 10 | 9 | 9 | 9 | 9 | 9 | 9 |
|  | *N_AE_* | 4.96 | 4.83 | 5.22 | 4.88 | 4.62 | 4.82 | 4.92 | 4.40 | 4.38 | 4.43 |
|  | *H_O_* | 0.63 | 0.73 | 0.77 | 0.77 | 0.70 | 0.66 | 0.67 | 0.56 | 0.66 | 0.59 |
|  | *H_E_* | 0.77 | 0.79 | 0.78 | 0.80 | 0.72 | 0.74 | 0.75 | 0.71 | 0.70 | 0.66 |
|  | *PIC* | 0.73 | 0.77 | 0.75 | 0.77 | 0.67 | 0.68 | 0.71 | 0.66 | 0.62 | 0.70 |
|  | *P_HWE_* | 0.51 | 0.97 | 0.90 | 0.07 | 0.29 | 0.97 | 0.99 | 0.08 | 0.12 | 0.14 |
| *Hier-ssr7* | *N_A_* | 10 | 11 | 11 | 11 | 10 | 9 | 8 | 9 | 10 | 9 |
|  | *N_AE_* | 5.17 | 6.92 | 5.03 | 7.12 | 5.32 | 4.47 | 4.16 | 4.82 | 5.05 | 4.86 |
|  | *H_O_* | 0.77 | 0.80 | 0.83 | 0.78 | 0.70 | 0.63 | 0.57 | 0.57 | 0.67 | 0.58 |
|  | *H_E_* | 0.76 | 0.86 | 0.80 | 0.86 | 0.75 | 0.78 | 0.67 | 0.75 | 0.78 | 0.68 |
|  | *PIC* | 0.73 | 0.84 | 0.77 | 0.85 | 0.71 | 0.71 | 0.61 | 0.78 | 0.76 | 0.75 |
|  | *P_HWE_* | 0.44 | 0.90 | 1.00 | 0.07 | 0.02* | 0.60 | 0.23 | 0.06 | 0.13 | 0.18 |
| *Hier-ssr8* | *N_A_* | 11 | 11 | 11 | 11 | 9 | 8 | 7 | 8 | 8 | 8 |
|  | *N_AE_* | 5.80 | 7.20 | 6.41 | 5.98 | 4.39 | 3.45 | 3.89 | 4.44 | 4.83 | 5.03 |
|  | *H_O_* | 0.77 | 0.83 | 0.77 | 0.83 | 0.80 | 0.79 | 0.81 | 0.80 | 0.83 | 0.83 |
|  | *H_E_* | 0.82 | 0.86 | 0.88 | 0.83 | 0.77 | 0.71 | 0.74 | 0.76 | 0.79 | 0.69 |
|  | *PIC* | 0.80 | 0.85 | 0.87 | 0.82 | 0.72 | 0.74 | 0.71 | 0.76 | 0.65 | 0.68 |
|  | *P_HWE_* | 0.17 | 0.95 | 0.08 | 0.24 | 0.50 | 0.32 | 0.54 | 0.11 | 0.80 | 0.92 |
| *Hier-ssr9* | *N_A_* | 10 | 10 | 10 | 10 | 10 | 10 | 9 | 10 | 10 | 10 |
|  | *N_AE_* | 5.66 | 4.66 | 4.41 | 5.27 | 4.17 | 3.74 | 4.04 | 5.20 | 5.53 | 4.64 |
|  | *H_O_* | 0.90 | 0.80 | 0.77 | 0.81 | 0.77 | 0.75 | 0.60 | 0.70 | 0.63 | 0.75 |
|  | *H_E_* | 0.80 | 0.75 | 0.75 | 0.81 | 0.74 | 0.71 | 0.75 | 0.81 | 0.80 | 0.78 |
|  | *PIC* | 0.77 | 0.71 | 0.72 | 0.74 | 0.78 | 0.70 | 0.72 | 0.80 | 0.70 | 0.68 |
|  | *P_HWE_* | 0.47 | 1.00 | 0.96 | 0.11 | 0.23 | 0.38 | 0.00** | 0.00** | 0.04* | 0.34 |
| *Hier-ssr10* | *N_A_* | 5 | 5 | 5 | 5 | 5 | 5 | 5 | 5 | 5 | 5 |
|  | *N_AE_* | 3.77 | 3.12 | 3.60 | 3.96 | 3.71 | 3.32 | 3.18 | 3.56 | 3.05 | 3.88 |
|  | *H_O_* | 0.73 | 0.77 | 0.77 | 0.69 | 0.50 | 0.56 | 0.62 | 0.67 | 0.74 | 0.63 |
|  | *H_E_* | 0.71 | 0.63 | 0.68 | 0.66 | 0.63 | 0.70 | 0.63 | 0.67 | 0.67 | 0.65 |
|  | *PIC* | 0.65 | 0.58 | 0.62 | 0.60 | 0.62 | 0.56 | 0.56 | 0.61 | 0.64 | 0.64 |
|  | *P_HWE_* | 0.74 | 0.84 | 0.83 | 0.79 | 0.54 | 0.48 | 0.50 | 0.06 | 0.81 | 0.23 |
| *Hier-ssr13* | *N_A_* | 8 | 8 | 7 | 7 | 8 | 7 | 7 | 7 | 7 | 7 |
|  | *N_AE_* | 4.86 | 3.81 | 4.56 | 4.43 | 4.27 | 3.47 | 3.90 | 4.61 | 4.70 | 4.04 |
|  | *H_O_* | 0.80 | 0.73 | 0.77 | 0.81 | 0.77 | 0.74 | 0.87 | 0.77 | 0.77 | 0.67 |
|  | *H_E_* | 0.79 | 0.74 | 0.72 | 0.77 | 0.69 | 0.71 | 0.74 | 0.62 | 0.79 | 0.75 |
|  | *PIC* | 0.76 | 0.71 | 0.68 | 0.74 | 0.57 | 0.66 | 0.70 | 0.76 | 0.72 | 0.67 |
|  | *P_HWE_* | 0.36 | 0.03 | 0.98 | 0.33 | 0.98 | 0.77 | 0.49 | 0.08 | 0.41 | 0.18 |
| *Hier-ssr15* | *N_A_* | 12 | 11 | 12 | 12 | 11 | 11 | 10 | 11 | 11 | 10 |
|  | *N_AE_* | 5.44 | 6.54 | 5.39 | 5.72 | 4.10 | 4.85 | 3.35 | 4.77 | 4.25 | 4.50 |
|  | *H_O_* | 0.83 | 0.89 | 0.86 | 0.89 | 0.77 | 0.79 | 0.67 | 0.87 | 0.76 | 0.75 |
|  | *H_E_* | 0.81 | 0.85 | 0.81 | 0.83 | 0.76 | 0.79 | 0.70 | 0.79 | 0.75 | 0.73 |
|  | *PIC* | 0.78 | 0.83 | 0.79 | 0.81 | 0.76 | 0.72 | 0.67 | 0.74 | 0.70 | 0.77 |
|  | *P_HWE_* | 0.37 | 0.30 | 0.86 | 0.62 | 0.93 | 0.00** | 0.49 | 0.07 | 0.12 | 0.08 |
| *Hier-ssr17* | *N_A_* | 9 | 8 | 9 | 9 | 8 | 8 | 8 | 9 | 9 | 9 |
|  | *N_AE_* | 6.03 | 5.51 | 5.36 | 5.74 | 5.59 | 5.62 | 4.95 | 4.93 | 4.83 | 5.72 |
|  | *H_O_* | 0.90 | 0.89 | 0.90 | 0.87 | 0.80 | 0.75 | 0.83 | 0.83 | 0.75 | 0.83 |
|  | *H_E_* | 0.83 | 0.78 | 0.77 | 0.83 | 0.78 | 0.85 | 0.79 | 0.78 | 0.78 | 0.84 |
|  | *PIC* | 0.80 | 0.75 | 0.74 | 0.76 | 0.75 | 0.75 | 0.76 | 0.75 | 0.82 | 0.83 |
|  | *P_HWE_* | 0.18 | 0.11 | 0.56 | 0.25 | 0.67 | 0.04* | 0.01* | 0.21 | 0.23 | 0.02* |
| *Hier-ssr28* | *N_A_* | 8 | 7 | 8 | 7 | 8 | 7 | 7 | 8 | 7 | 8 |
|  | *N_AE_* | 5.74 | 5.16 | 5.64 | 4.78 | 4.62 | 4.52 | 4.86 | 4.26 | 4.29 | 4.28 |
|  | *H_O_* | 0.83 | 0.69 | 0.72 | 0.73 | 0.80 | 0.73 | 0.70 | 0.70 | 0.77 | 0.73 |
|  | *H_E_* | 0.82 | 0.81 | 0.81 | 0.79 | 0.73 | 0.79 | 0.79 | 0.77 | 0.74 | 0.77 |
|  | *PIC* | 0.79 | 0.78 | 0.78 | 0.76 | 0.73 | 0.61 | 0.77 | 0.73 | 0.74 | 0.78 |
|  | *P_HWE_* | 0.09 | 0.05 | 0.62 | 0.05 | 0.38 | 0.23 | 0.31 | 0.96 | 0.03* | 0.10 |
| *Hier-ssr29* | *N_A_* | 11 | 11 | 11 | 10 | 11 | 11 | 10 | 11 | 10 | 11 |
|  | *N_AE_* | 5.68 | 6.06 | 5.54 | 4.60 | 4.87 | 5.34 | 4.69 | 4.66 | 3.55 | 3.78 |
|  | *H_O_* | 0.87 | 0.87 | 0.75 | 0.76 | 0.87 | 0.81 | 0.68 | 0.67 | 0.69 | 0.73 |
|  | *H_E_* | 0.73 | 0.84 | 0.78 | 0.78 | 0.77 | 0.81 | 0.63 | 0.77 | 0.72 | 0.70 |
|  | *PIC* | 0.73 | 0.82 | 0.75 | 0.75 | 0.73 | 0.74 | 0.59 | 0.69 | 0.72 | 0.82 |
|  | *P_HWE_* | 0.40 | 0.06 | 0.92 | 0.53 | 0.10 | 0.21 | 0.73 | 0.33 | 0.90 | 0.07 |
| *Hier-ssr51* | *N_A_* | 8 | 8 | 8 | 8 | 8 | 8 | 8 | 8 | 8 | 8 |
|  | *N_AE_* | 5.11 | 4.89 | 5.35 | 5.47 | 4.90 | 4.37 | 5.61 | 5.63 | 6.14 | 5.26 |
|  | *H_O_* | 0.83 | 0.80 | 0.83 | 0.86 | 0.90 | 0.84 | 0.83 | 0.87 | 0.73 | 0.82 |
|  | *H_E_* | 0.79 | 0.80 | 0.77 | 0.82 | 0.80 | 0.77 | 0.82 | 0.82 | 0.82 | 0.81 |
|  | *PIC* | 0.76 | 0.77 | 0.74 | 0.79 | 0.80 | 0.77 | 0.80 | 0.82 | 0.78 | 0.75 |
|  | *P_HWE_* | 0.41 | 0.37 | 0.05 | 0.25 | 0.74 | 0.65 | 0.56 | 0.12 | 0.08 | 0.20 |

*N_A_*, number of alleles at each locus; *N_AE_*, number of effective alleles; *H_O_*, observed heterozygosity; *H_E_*, expected heterozygosity; *P_HWE_*, estimated probability of Hardy-Weinberg equilibrium; *, significant deviation from Hardy-Weinberg equilibrium (*p* < 0.05).
