# Supplementary material for: Potential Risks for Seahorse Stock Enhancement: Insight From the Declivity of Genetic Levels With Hatchery Management
Source: Front Genet. 2022 Jan 20;12:830626. doi: 10.3389/fgene.2021.830626 (PMC8811164; doi:10.3389/fgene.2021.830626)
Supplement: Supplementary file 4 [file Table4.DOCX]

**Table S3** Hierarchical analysis of molecular variance (AMOVA) of all lined seahorse (*Hippocampus erectus*) collections

| Source of variation | d.f. | Sum of squares | Variance of components | Percentage of variation |
| --- | --- | --- | --- | --- |
| Among populations | 9 | 221.98 | 0.33 | 7.35 |
| Within populations | 610 | 2544.22 | 4.17 | 92.65 |
| Total | 619 | 2766.20 | 4.50 | 100.00 |
